# Supplementary figures and images for: Impact of processing method on donated human breast milk microRNA content
Source: PLoS One. 2020 Jul 15;15(7):e0236126. doi: 10.1371/journal.pone.0236126 (PMC7363072; doi:10.1371/journal.pone.0236126)

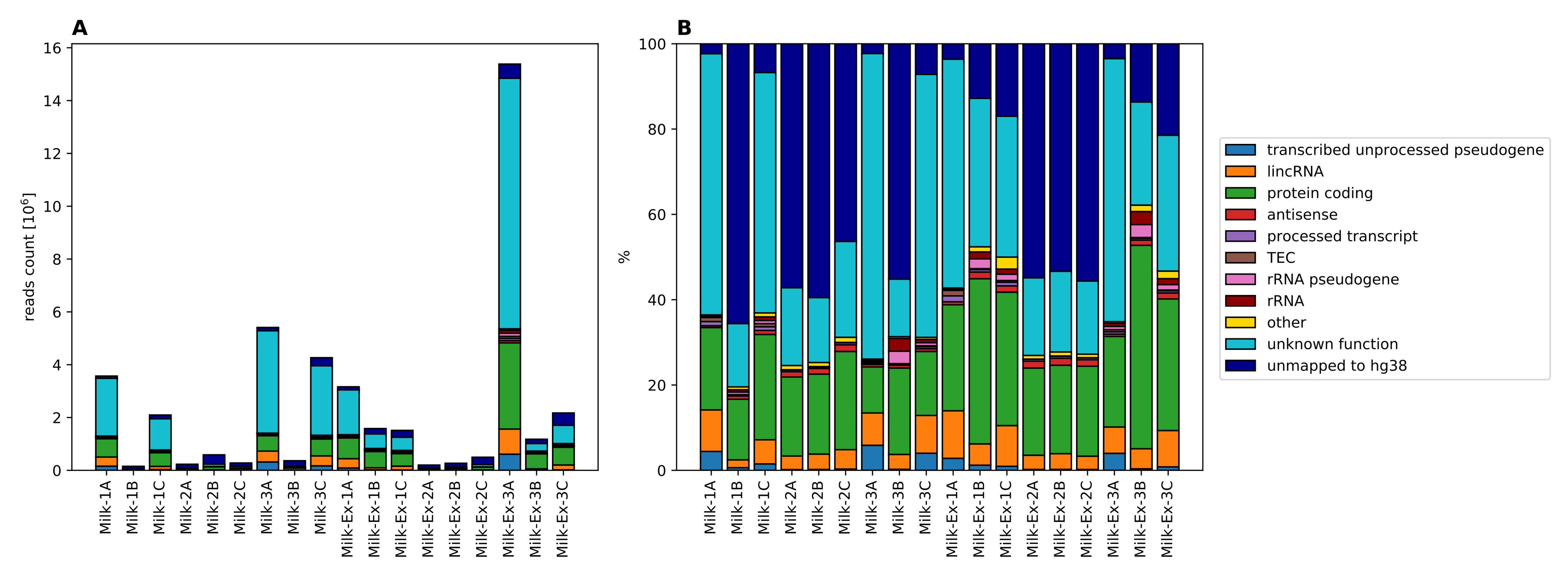

Supplement: S1 Fig — (TIF) [file pone.0236126.s001.tif]
