# Supplementary material for: Impact of processing method on donated human breast milk microRNA content
Source: PLoS One. 2020 Jul 15;15(7):e0236126. doi: 10.1371/journal.pone.0236126 (PMC7363072; doi:10.1371/journal.pone.0236126)
Supplement: S1 Table — (DOCX) [file pone.0236126.s002.docx]

**Supplementary Table S1:** Samples data. cDNA concentration was measured by Quibit fluorometer twice before library normalization and a mean of 2 measurements was inserted into table. A read was considered as mapped only if it mapped uniquely.

| **Sample name** | **Donor** | **Proceesing method** | **Material for RNA extraction** | **cDNA concentration [ng/µl]** | **Number of reads** | **Reads mapped to miRBase** |
| --- | --- | --- | --- | --- | --- | --- |
| Milk-1A | A | None | whole | 4.00 | 7083244 | 271670 |
| Milk-1B | B | None | whole | 0.064 | 3985727 | 6340 |
| Milk-1C | C | None | whole | 3.93 | 5580417 | 544295 |
| Milk-2A | A | HoP | whole | 0.501 | 3073433 | 4246 |
| Milk-2B | B | HoP | whole | 0.442 | 3481401 | 5334 |
| Milk-2C | C | HoP | whole | 0.725 | 3465824 | 5736 |
| Milk-3A | A | HPP | whole | 13.55 | 7520075 | 111099 |
| Milk-3B | B | HPP | whole | 0.165 | 4355459 | 3087 |
| Milk-3C | C | HPP | whole | 5.75 | 7733423 | 84785 |
| Milk-Ex-1A | A | None | exosomes | 11.85 | 7993211 | 2037278 |
| Milk-Ex-1B | B | None | exosomes | 19.2 | 7608741 | 1004479 |
| Milk-Ex-1C | C | None | exosomes | 0.539 | 5933260 | 508353 |
| Milk-Ex-2A | A | HoP | exosomes | 1.70 | 3367403 | 4618 |
| Milk-Ex-2B | B | HoP | exosomes | 1.20 | 4209211 | 4436 |
| Milk-Ex-2C | C | HoP | exosomes | 1.75 | 6718926 | 2686 |
| Milk-Ex-3A | A | HPP | exosomes | 1.66 | 30512466 | 2148668 |
| Milk-Ex-3B | B | HPP | exosomes | 1.405 | 7168949 | 141038 |
| Milk-Ex-3C | C | HPP | exosomes | 1.575 | 10921017 | 270943 |
