# Supplementary material for: Impact of processing method on donated human breast milk microRNA content
Source: PLoS One. 2020 Jul 15;15(7):e0236126. doi: 10.1371/journal.pone.0236126 (PMC7363072; doi:10.1371/journal.pone.0236126)
Supplement: S4 Table — (DOCX) [file pone.0236126.s005.docx]

**Supplementary Table S2:** The most abundant miRNA accounting for 90% of whole material and exosomal reads in unprocessed milk.

| **Material** | **miRNA** |
| --- | --- |
| Whole | miR-148a-3p, miR-29c-3p, miR-29a-3p, let-7b-5p, miR-30d-5p, miR-200a-3p, miR-141-3p, miR-200c-3p, let-7a-5p, miR-200b-3p, miR-22-3p, miR-30a-5p, miR-26a-5p, miR-191-5p, miR‑16-5p, miR-375-3p, miR-30b-5p, miR-148b-3p, let-7f-5p, miR-423-5p, miR-146b-5p, let‑7g-5p, miR-320a-3p, miR-30c-5p, miR-182-5p, let-7i-5p, miR-183-5p, miR-25-3p, miR‑125a‑5p, miR-365a-3p, miR-365b-3p, miR-92a-3p, let-7c-5p, miR-30e-5p, miR-21-5p, miR-429, miR-125b-5p, miR-101-3p, miR-99a-5p, miR-29b-3p, miR-205-5p |
| Exosomal​ | miR-148a-3p, miR-141-3p, miR-29c-3p, let-7b-5p, miR-29a-3p, miR-200c-3p, miR-30d-5p, miR-200a-3p, let-7a-5p, miR-26a-5p, miR-375-3p, miR-30b-5p, miR-16-5p, let-7f-5p, miR 146b-5p, miR-21-5p, miR-423-5p, miR-30a-5p, miR-200b-3p, miR-22-3p, miR-182-5p, miR-191-5p, let-7c-5p, miR-92a-3p, miR-320a-3p, miR-429, let-7g-5p, let-7i-5p, miR-183-5p, miR-181a-5p, miR-26b-5p, miR-99a-5p, miR-30e-5p, miR-30c-5p, miR-205-5p |
